# Transcutaneous spinal stimulation in patients with intrathecal baclofen pump delivery system: A preliminary safety study

**DOI:** 10.3389/fnins.2022.1075293

**Published:** 2022-12-21

**Authors:** John Lopez, Gail F. Forrest, Einat Engel-Haber, Brittany Snider, Kam Momeni, Manikandan Ravi, Steven Kirshblum

**Affiliations:** ^1^Kessler Institute for Rehabilitation, West Orange, NJ, United States; ^2^New Jersey Medical School, Rutgers, The State University of New Jersey, Newark, NJ, United States; ^3^Kessler Foundation, West Orange, NJ, United States; ^4^Koneksa Health, New York, NY, United States

**Keywords:** transcutaenous spinal cord stimulation, intrathecal baclofen pump, spinal cord injury, spasticity, spinal stimulation

## Abstract

**Objective:**

To determine the effect of transcutaneous spinal stimulation (TSS) on an implanted intrathecal baclofen (ITB) pump in persons with traumatic spinal cord injury (SCI).

**Design:**

Prospective clinical trial.

**Participants:**

Five individuals with chronic traumatic SCI, >18 years of age, and an anteriorly implanted Medtronic SynchroMed™ II ITB pump delivery system.

**Intervention:**

Transcutaneous spinal stimulation trials with cathode at T11/12, with pump interrogation before, during and after stimulation.

**Results:**

There was no evidence of any effect of the TSS in regards to disruption of the ITB pump delivery mechanism. Communication interference with the interrogator to the pump occurred often during stimulation for log transmission most likely secondary to the electromagnetic interference from the stimulation. One individual had elevated blood pressure at the end of the trial, suspected to be unrelated to the spinal stimulation.

**Conclusion:**

Based upon this pilot study, further TSS studies including persons with an implanted Medtronic SynchroMed™ II ITB pump can be considered when stimulating at the low thoracic spine, although communication with the programmer during the stimulation may be affected.

## Introduction

Traumatic spinal cord injury (SCI) has an incidence of approximately 17,900 cases per year with an estimated prevalence ranging from 252,000 to 373,000 persons in the United States (National Spinal Cord Injury Statistical Center, 2021). SCI can result in lifelong impairments and varying levels of functional compromise. Spasticity is a common secondary medical complication associated with SCI that affects up to 65% of patients with SCI (Holtz et al., 2017, 2018; Mills et al., 2020; Richard-Denis et al., 2020). Spasticity most often negatively impacts physical health, functional activities, as well as quality of life; affecting domains of social, vocational, and psychological wellbeing (Post et al., 1998; Mahoney et al., 2007; Westerkam et al., 2011; Andresen et al., 2016; Barnes et al., 2017; Tibbett et al., 2020; Vural et al., 2020; Field-Fote et al., 2022). Numerous treatment strategies are available for the treatment of spasticity including modalities, medications, botulinum toxin injections, and intrathecal baclofen (ITB) pump delivery systems for persons in whom spasticity does not otherwise respond to these interventions (Walker et al., 2016).

Transcutaneous spinal stimulation (TSS), a novel treatment strategy that utilizes surface electrodes to generate currents across the vertebral canal affecting spinal segments, can potentially improve motor activity and enhance function (Hofstoetter et al., 2013; Gad et al., 2017, 2018; Inanici et al., 2018; Sayenko et al., 2019; Al’joboori et al., 2020; Meyer et al., 2020; Zhang et al., 2020), as well as decrease secondary effects of the injury including stabilizing blood pressure (BP) (Phillips et al., 2018), and decreasing spasticity (Dimitrijevic M. M. et al., 1986; Dimitrijevic M. R. et al., 1986; Hofstoetter et al., 2014, 2020; Estes et al., 2017). TSS has also been effective in decreasing spasticity following multiple sclerosis (Hofstoetter et al., 2021).

Despite the potential benefits of treatment with TSS for the SCI population, no studies to date have assessed the interaction between TSS and ITB pumps, as patients with implantable systems are often excluded from TSS trials (Estes et al., 2017; Al’joboori et al., 2020; Hofstoetter et al., 2020; Meyer et al., 2020). Patients with severe spasticity that necessitated an ITB pump for treatment, may often have other impairments that may benefit from the innovative intervention of TSS.

This study was undertaken to determine the effect of moderate intensity spinal stimulation on the implanted ITB pump and delivery of the medication in persons with traumatic SCI. We hypothesized that this type of spinal stimulation would not have a negative impact on the delivery of the intrathecal pump medication.

## Materials and methods

### Design

This prospective study was approved by our local institutional review board prior to enrollment. Eligible participants included individuals with traumatic SCI >6 months who had an anteriorly implanted ITB pump delivery system <7 years prior for management of spasticity, ≥18 years of age, and a neurologic level of injury (NLI) above T12. Exclusion criteria included history of seizure disorder or malignancy, current pregnancy, and magnetic resonance imaging (MRI) within the past 24 h, and ITB pump interrogation or refill within the past 24 h.

After informed consent was completed, each participant completed two separate stimulation trials. For stimulation, each subject was placed in the supine position, arms and hands pronated, and shoes removed. Self-adhesive round stimulating electrodes (STIMEX, schwa-medico GmbH, Germany) were applied over the skin at the midline of spinous processes as cathodes (T11/12) and a pair of rectangular anode electrodes (8 cm × 13 cm) were placed over the iliac crests. Surface electromyography (sEMG) electrodes were placed on bilateral rectus femoris, vastus lateralis, tibialis anterior, and gastrocnemius muscles. sEMG data were collected at 10,000 Hz using the MA400 systems (Motion Lab Systems, Inc., Baton Rouge, LA, USA). The first trial was used to determine stimulation intensity (BioStim-5, Cosyma, Russia) where evoked potentials were present on the recruitment curve in at least six out of eight of the lower extremity muscles.

One spinal site (T11/12) was tested for initial amplitude response and with the stimulation frequency of 2 Hz (monophasic, rectangular 1-ms pulses with a carrier-wave frequency set at 5 kHz). Stimulation started at 5 mA and increased by 5 mA increments (Zhang et al., 2020). The second trial involved continuous TSS over a 30-min period based on parameters determined from the first trial. Sub-threshold, tonic stimulation was delivered at dorsal spinal segments (at T11/12) over the skin using 30 Hz, monophasic, rectangular 1-ms pulses with a carrier-wave frequency set at 5 kHz, with intensities between 40 and 60 mA.

The pump was interrogated 10 min prior to initiation of stimulation, immediately prior to stimulation, every 5 min until completion of stimulation, and 30 min following discontinuation of stimulation. BP was monitored via a brachial artery BP cuff every 5 min throughout the entire study, and was taken at any additional time point if symptoms of autonomic dysreflexia were noted.

### Outcome measures

Assessment of pump delivery malfunction, *via* ITB pump interrogation and review of event logs, was the primary outcome measure to determine if motor stall or breakdown occurred. Adverse events were assessed throughout the trials and BP measurements were obtained to monitor for autonomic dysreflexia. Participants completed a Global Impression of Change scale at the completion of stimulation.

Participants were contacted 1 day after the stimulation to identify subjective changes in tone or pain. Additionally, the next refill for each participant was monitored for any abnormalities, such as a volume discrepancy (between the measured and expected pump reservoir fluid volumes) outside the manufacturer’s accuracy specification.

## Results

During the study period, seven individuals with an implanted Medtronic SynchroMed™ II ITB pump were screened and five participants completed the spinal stimulation protocol. The two subjects who did not meet final criteria were excluded due to the presence of a medical condition (malignancy and ongoing treatment for an active infection). Demographic data for the active participants are listed in Table 1.

**TABLE 1:** Demographic data of subjects.

Participant	Sex	Age (years)	NLI	AIS grade
1	M	51	C5	B
2	M	58	C3	D
3	M	36	C5	A
4	M	36	C6	B
5	M	32	T1	C

M, male; NLI, neurological level of injury; AIS, American Spinal Injury (ASIA) impairment scale.

There was no evidence in any of the cases of any disruption from the TSS on the SynchroMed™ II pump delivery mechanism as documented by the interrogation scans during or after the stimulation period. An interesting and repeatable finding in each case was that the communication between the interrogator and pump for log transmission was inconsistent during TSS. Specifically, for each subject throughout the stimulation period, multiple attempts of communication of the programmer interfacing with the interrogator failed, whereas prior to and after each session there was no delay in interrogation. However, in each case, after a number of attempts (usually 3–5), the interrogation was successfully completed.

Four participants had no adverse events during or after the study intervention. One individual (Subject # 4) developed elevated BP meeting criteria for autonomic dysreflexia [defined as a rise in systolic BP >20 mmHg above baseline (Krassioukov et al., 2021)], after 25 min of stimulation (second trial). Peak BP during this episode was 198/112 mmHg. After sitting the patient upright and performing intermittent catheterization, the BP steadily decreased and at 1 h post catheterization was 150/77 mmHg. During the episode of autonomic dysreflexia, the participant described symptoms similar to those he experienced during previous urinary tract infections, including sensations of bladder spasms and groin pain radiating to his left lower extremity. As the BP improved, the symptoms resolved. Further evaluation revealed a urinary tract infection based upon symptoms, urinalysis, culture and sensitivity, and the participant was treated with antibiotics by his primary SCI physician.

One other participant had one asymptomatic systolic BP reading that was >20 mmHg above baseline on an isolated reading (an elevation from 101/63 mmHg at baseline to 132/74 mmHg, without a significant change in heart rate) which resolved by the next BP reading (116/72 mmHg) without intervention or interruption. BP readings for the other subjects remained stable throughout the study period.

After each of the trials, the four subjects who did not experience the episode of autonomic dysreflexia as noted above had no change noted on their Global Impression of Change at the completion of stimulation. At follow-up for routine pump refill, no participant experienced objective increases in tone or required ITB pump dose adjustments. Additionally, no abnormalities appeared in the ITB pump event log.

## Discussion

To our knowledge, this is the first prospective evaluation of ITB pump delivery in persons with SCI undergoing TSS. During the stimulation trials, no participant had any SynchroMed™ II ITB pump malfunction evidenced by pump log evaluation. This was consistent with our hypothesis that the pump would not be stalled or damaged by the stimulation. The design and construction (e.g., titanium shield) of the pump as well as its implant location away from the TSS may contribute to its resilience. While there was one adverse event noted during the trial, it was most likely unrelated to the interaction of the spinal stimulation and the baclofen pump, and instead due to bladder related issues including a urinary tract infection possibly combined with the supine position during the trial.

The interesting and repeatable finding was the interference of the communication between the interrogator and the pump, as log transmission was affected during TSS. The probable reason for the interruption is the electronic field created by the TSS [a form of electromagnetic interference (EMI) creating obstruction with the programmer telemetry]. As noted in the labeling of the intrathecal pump, “EMI can interfere with programmer telemetry. If EMI disrupts programming, move the programmer away from the likely source of the EMI (SynchroMed™ IsoMed™, 2020, page 17).” Moving the interrogator off the skin was at times helpful in decreasing the interference, although was not effective in all cases, as it is recommended to be a minimum of 20 cm away from the source to minimize telemetry interference (SynchroMed™ IsoMed™, 2020, page 16).

There are a few previous studies that have demonstrated the safety of the combined neuromodulation approach of an implanted spinal cord stimulator and an intrathecal pump (e.g., intrathecal baclofen, clonidine, or opioids) for treatment of neuropathic pain, complex regional pain syndrome, and failed back syndrome (Lind et al., 2004, 2008; Schechtmann et al., 2010; Tomycz et al., 2010; Goto et al., 2013). This study, however, is the first we are aware of to address the pump mechanism during stimulation with TSS in persons with SCI.

There are some limitations to this initial pilot study. Only one stimulation paradigm was completed in this trial. It was felt that the effect on the pump mechanism would not be different based upon changes in stimulation parameters, although further study may be needed. The main objective of this trial was to study the safety of TSS in general in persons with an ITB pump in place. Moreover, this study was the first to evaluate the effect of spinal stimulation on the pump mechanism for SCI. Therefore, the investigators implemented a conservative protocol with a moderate stimulation level. Since communication interference was observed even at this intensity, the effect of higher stimulation intensities on the pump should be evaluated in future studies.

Additionally, we did not study the effectiveness of the stimulation parameters from a physiological or functional standpoint, e.g., decreasing the level of spasticity or enhancing motor control, and the study of these important issues would be performed at a later time once deemed safe. Lastly, only one type of intrathecal pump (Medtronic SynchroMed™ II) was studied, although this was not an exclusion criteria.

Based upon this pilot trial, TSS may be performed safely in persons with an implanted Medtronic SynchroMed™ II ITB pump when stimulating at the thoracic spine (T11/12), although communication with the programmer during the stimulation may be affected due to near field interference. One subject developed autonomic dysreflexia during the trial that was not deemed to be secondary to the effect of the stimulation with the pump in place. Further incremental research is warranted to evaluate greater intensity of stimulation with modulation in frequency and waveform. In addition, further research is needed to study the effect of multi-site stimulation on pump function, as well as the impact of repeated sessions.

## Data availability statement

The original contributions presented in this study are included in this article/supplementary material, further inquiries can be directed to the corresponding author.

## Ethics statement

The studies involving human participants were reviewed and approved by the Institutional Review Board at Kessler Foundation. The patients/participants provided their written informed consent to participate in this study.

## Author contributions

JL and SK contributed to the conception and design of the study. JL, GF, KM, MR, SK completed the acquisition of data. JL initiated the first draft of the manuscript. SK, GF, EE-H, and BS wrote sections of the manuscript. All authors contributed to manuscript revision, read, and approved the submitted version.
